# Supplementary material for: Androgen Deprivation-Induced TET2 Activation Fuels Prostate Cancer Progression via Epigenetic Priming and Slow-Cycling Cancer Cells
Source: bioRxiv. 2025 Mar 29:2025.03.26.645495. Preprint. [Version 1] doi: 10.1101/2025.03.26.645495 (PMC11974783; doi:10.1101/2025.03.26.645495)

bioRxiv preprint doi: https://doi.org/10.1101/2025.03.26.645495; this version posted March 29, 2025. The copyright holder for this preprint (which was not certified by peer review) is the author/funder, who has granted bioRxiv a license to display the preprint in perpetuity. It is made available under aCC-BY 4.0 International license.

Supplementary Figure 1. AR directly binds to the cis-regulatory sequence of TET2. CHIPseq and ATACseq data from LNCaP cells cultured with or without androgen illustrating distinct binding patterns of various factors at the TET2 locus. Red arrows indicate increased H3K27ac peaks following androgen deprivation, suggesting activation of this region. Blue arrows indicate reduced AR binding peaks after androgen removal.

Supplementary Figure 2. Validation of TET2 knockdown efficiency in PC3 cells. TET2 knockdown efficiency was confirmed by RT-qPCR in PC3 cells. *Student t-test,* $p < 0.05$.

bioRxiv preprint doi: https://doi.org/10.1101/2025.03.26.645495; this version posted March 29, 2025. The copyright holder for this preprint (which was not certified by peer review) is the author/funder, who has granted bioRxiv a license to display the preprint in perpetuity. It is made available under aCC-BY 4.0 International license.

Supplementary Figure 3. TET2 regulates the expression of cell cycle- and DDR-related genes. RT-qPCR analyses of cell cycle-related genes in PC3 GFP and TET2KD cells cultured in full serum media, C4-2B GFP and TET2KD cells cultured in ADT conditions, n=3. *Student t-test,* $p < 0.05$.

bioRxiv preprint doi: https://doi.org/10.1101/2025.03.26.645495; this version posted March 29, 2025. The copyright holder for this preprint (which was not certified by peer review) is the author/funder, who has granted bioRxiv a license to display the preprint in perpetuity. It is made available under aCC-BY 4.0 International license.

sFig.1

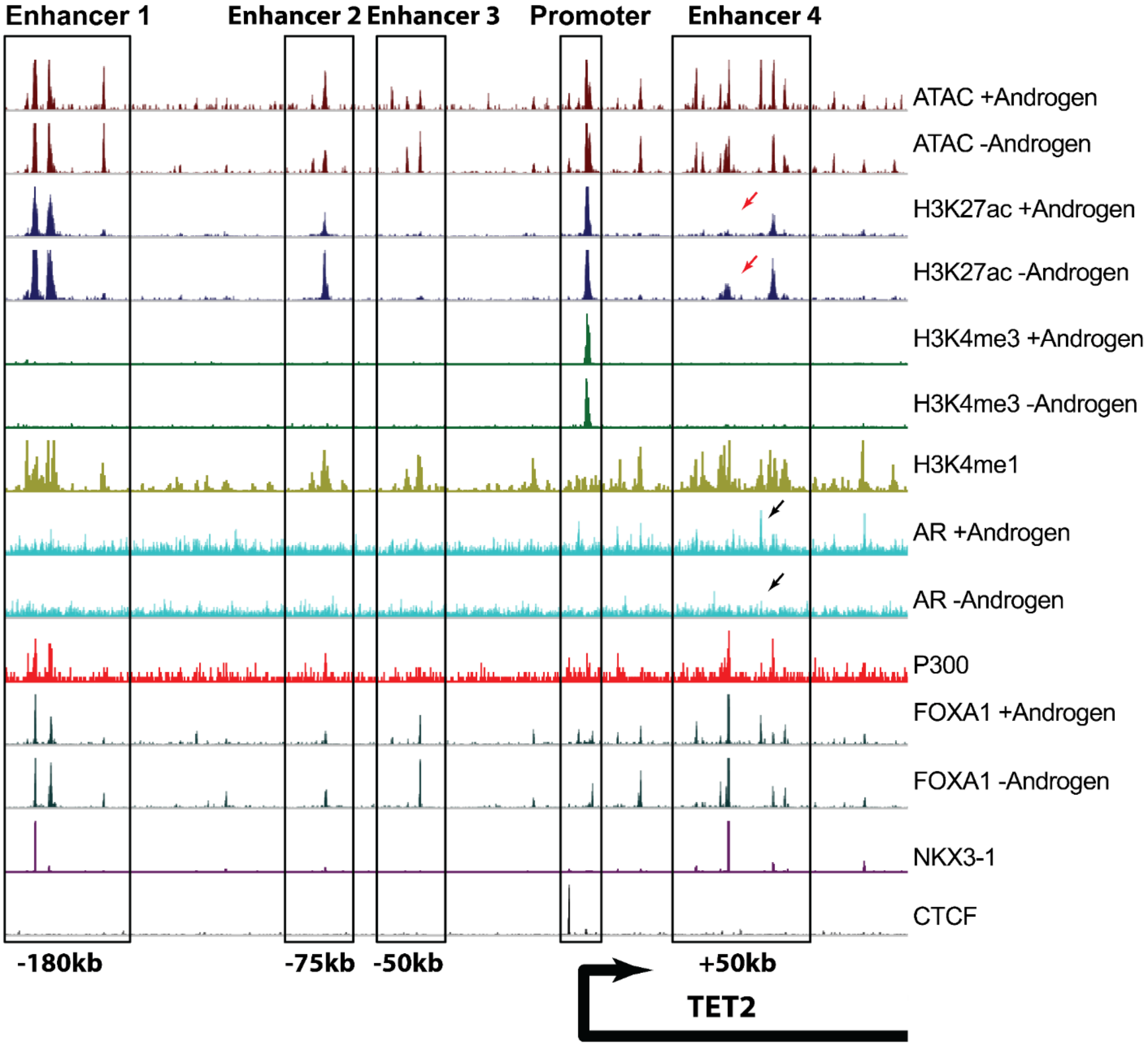

bioRxiv preprint doi: https://doi.org/10.1101/2025.03.26.645495; this version posted March 29, 2025. The copyright holder for this preprint (which was not certified by peer review) is the author/funder, who has granted bioRxiv a license to display the preprint in perpetuity. It is made available under aCC-BY 4.0 International license.

sFig. 2

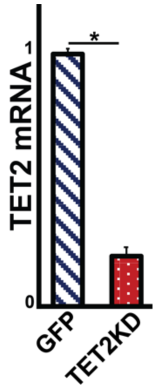

bioRxiv preprint doi: https://doi.org/10.1101/2025.03.26.645495; this version posted March 29, 2025. The copyright holder for this preprint (which was not certified by peer review) is the author/funder, who has granted bioRxiv a license to display the preprint in perpetuity. It is made available under aCC-BY 4.0 International license.

sFig.3

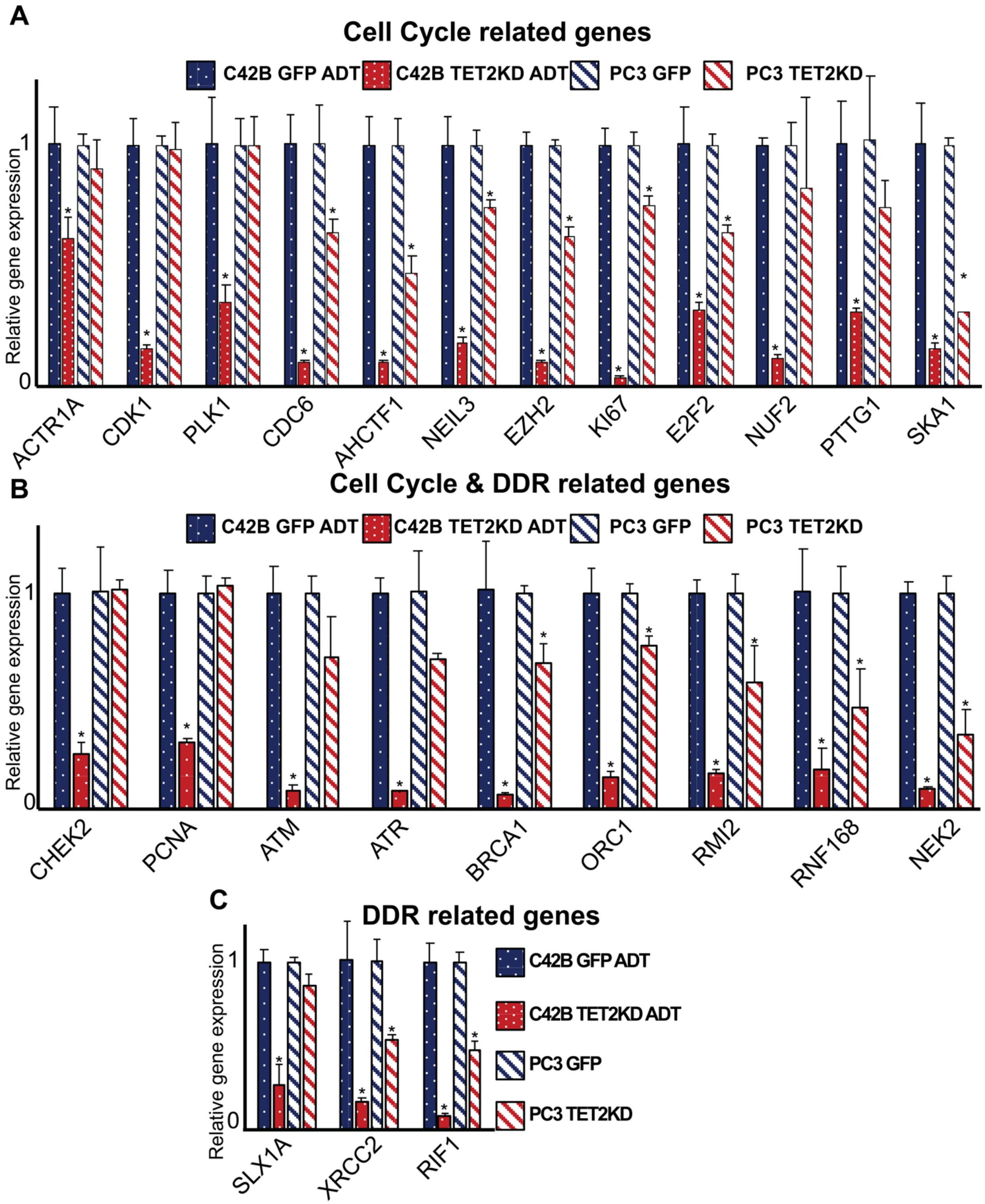

Supplement: Supplement 1 [file NIHPP2025.03.26.645495v1-supplement-1.pdf]
